# Assessment of angiopoietin-1, angiopoietin-2, Tie-2, EphrinB2, EphB4, and endocan serum level in patients with Behçet disease: A cross-sectional observational study

**DOI:** 10.1097/MD.0000000000048261

**Published:** 2026-04-24

**Authors:** Betül Gökçek Barutcigil, Emine Çinici, Esra Laloglu

**Affiliations:** a Department of Ophthalmology, Tekirdağ City Hospital, Tekirdağ, Türkiye; b Department of Ophthalmology, Atatürk University School of Medicine, Erzurum, Türkiye; c Department of Clinical Biochemistry, Atatürk University School of Medicine, Erzurum, Türkiye.

**Keywords:** angiopoietin-1 (Ang-1), angiopoietin-2 (Ang-2), Behçet disease, endocan, endothelial dysfunction, EphB4, EphrinB2, Tie-2, uveitis, vascular inflammation

## Abstract

Behçet disease (BD) is an inflammatory disorder with significant ocular involvement. Angiopoietins regulate vascular stability, while EphrinB2/EphB4 interactions are essential for retinal neovascularization and endothelial behavior. Endocan, a marker of endothelial activation, contributes to inflammatory processes by mediating leukocyte migration. This cross-sectional study investigates the role of angiopoietins, EphrinB2/EphB4 signaling, and endocan in the development of active uveitis in BD patients. We recruited 29 BD patients with active eye involvement (group 1), 31 BD patients without eye involvement (group 2), and 30 healthy controls (group 3). Serum tyrosine protein kinase receptor (Tie-2), angiopoietin-1 (Ang-1), angiopoietin-2 (Ang-2), EphrinB2, EphB4, and endocan levels were determined by the enzyme-linked immunosorbent assay method. A statistically significant difference was found between the Ang-1, Ang-2, Tie-2, EphrinB2, EphB4, and endocan levels of the 3 different groups in the study (*P* < .05). Between the patients with panuveitis and patients with only posterior or anterior uveitis, serum Ang-1 (*P* < .001), Tie-2 (*P* < .001), EphrinB2 (*P* < .001), EphB4 (*P* = .001), and endocan (*P* = .002) levels had statistically significant differences. The highest and significant correlation was found between EphrinB2 and EphB4 (*r* = 0.931, *P* < .001). This was followed by a high level of significant correlation between Ang-1 and Ang-2 (*r* = −0.845, *P* < .001). In the group with active uveitis, EphB4 (*r* = −0.774), Ang-2 (*r* = −0.763), and Tie-2 (*r* = −0.701) were negatively and highly significantly correlated with best corrected visual acuity (*P* < .001). Ang-1, Ang-2, Tie-2, EphrinB2, EphB4, and endocan regulate vascular stability, retinal neovascularization, and inflammation, potentially contributing to BD-associated uveitis. These findings could have important implications for the diagnosis and management of ocular involvement of BD.

## 1. Introduction

Behçet disease (BD) is an inflammatory disorder described by Dr Hulusi Behçet and characterized by vasculitis with recurrent oral aphthae, genital ulcers, uveitis, gastrointestinal, and central nervous system involvement.^[1]^ The etiology of BD is not fully understood, but immune defects and genetic inheritance are among the strong hypotheses discussed.^[2]^ The disease can affect the deep vein system, causing thrombophlebitis, as well as the pulmonary, cardiac, and nervous systems. The role of some immune system cells and the pro-inflammatory cytokines they release in the rapid deterioration of vascular endothelial function in BD has been emphasized.^[3]^

Ocular manifestations affect approximately 70% of BD patients. It is characterized by chronic and recurrent attacks and is unilateral in 20% and bilateral in 80% of patients. It has a rapid and severe prognosis at young ages and in the male patient group.^[4]^

The most common ocular manifestations are chronic recurrent non-granulomatous uveitis and necrotizing obliterative retinal vasculitis, which can cause severe damage to intraocular structures. It is observed as occlusive necrotizing vasculitis affecting both arteries and veins.

Angiopoietins are protein-structured growth factors that regulate angiogenesis. Angiopoietin-1 (Ang-1) is a molecule that binds to the tyrosine protein kinase receptor (Tie-2) and functions in physiological structure. Ang-1 is secreted by periendothelial cells and platelet α-granules. Angiopoietin-2 (Ang-2) acts as an antagonist to Ang-1. In pathological conditions, it induces angiogenesis and causes vascular destabilization.^[5]^ It also makes blood vessels more sensitive to vascular endothelial growth factor A.^[6]^ Tie-2 is a cell surface tyrosine kinase receptor to which angiopoietins bind. High activation of Tie-2 in quiescent, non-angiogenic blood vessels is extremely important for the protection of the vascular barrier. While the Ang-1/Tie-2 pathway works in physiological balance, Ang-2 is released with any pathology and binds to the Tie-2 receptor. Agonistic Ang-1 functions are antagonized by Ang-2.^[7]^ It does not phosphorylate the receptor and performs inhibition.

The complementary expression of EphrinB2 and its receptor EphB4 on embryonic arteries and veins, respectively, suggests a reciprocal interaction in vascular remodeling.^[8]^ During embryogenesis, both EphrinB2 and EphB4 have been shown to play pivotal roles in vasculogenesis.^[9]^ In vitro studies have demonstrated that human retinal neovascular membrane endothelial cells express EphrinB2 and EphB4, further supporting the role of these molecules in retinal endothelial cell biology.^[10]^ Under inflammatory conditions, Ephrin proteins induce phenotypic changes in the vascular endothelium, thereby facilitating the transmigration of inflammatory cells across the vessel wall.^[8]^ Furthermore, the activation of Ephrin signaling pathways has been reported following ischemia/reperfusion injury, suggesting a role in the endothelial response to tissue hypoxia and oxidative stress.^[11]^

While EphB4 causes differentiation in venous endothelial cells, EphrinB2 mostly causes differentiation in arterial structures.^[12]^ Yuuki et al reported that EphB4/EphrinB2 play a key role in neovascularization developing in ocular structures.^[9]^

Endocan, formerly known as the human endothelial cell-specific molecule, is released from endothelial cells as a soluble proteoglycan. It is a biomarker of endothelial cell activation.^[13]^ Endocan binds to leukocytes via lymphocyte function antigen-1 (LFA-1) and impairs LFA-1-mediated leukocyte function by inhibiting the LFA-1/intercellular adhesion molecule 1 junction.^[14]^ In cases of systemic inflammation and sepsis, it has been shown that there is a correlation between disease severity and endocan levels.^[15]^

Recent studies have demonstrated the importance of identifying biomarkers and imaging-based tools that can specifically localize and delineate ocular pathology, thereby enabling retina specialists to accurately diagnose and optimize treatment strategies in conditions such as diabetic macular edema and neovascular age-related macular degeneration.^[16,17]^

Despite advances in treatment, recurrent uveitis remains a serious problem in ophthalmological practice. There are currently no validated serum biomarkers in routine clinical use for the early detection of ocular involvement or the assessment of disease severity in BD. The aim of this study was to find the role of these markers that are associated with endothelial dysfunction in the development of active uveitis in BD patients.

The identification of such biomarkers would signify a substantial advancement in the clinical management of BD-related uveitis on a global scale.

## 2. Method

### 2.1. Patient selection

This study was carried out at the Ophthalmology and Dermatology Outpatient Clinics of Atatürk University Faculty of Medicine Hospital. Ethical approval was obtained from the Atatürk University Ethics Committee for Clinical Research (Decision No: 2022/58), and all procedures adhered to the principles of the Declaration of Helsinki. BD patients aged 18 to 60 years are included. The study included 29 BD patients with active eye involvement (group 1) and 31 BD patients without eye involvement (group 2). Thirty volunteer and healthy individuals with similar sociodemographic characteristics (group 3) were taken as the control group. Patients were questioned in terms of age, gender, comorbidity, onset date of complaints, duration of the disease, disease involvement, and medications used. All patients included in the study underwent a detailed ophthalmological evaluation, including best corrected visual acuity (BCVA), eye pressure, anterior segment, and fundus examinations, in the Ophthalmology Outpatient Clinic. Patients with active uveitis were evaluated in the first week of the attack, and blood samples were taken before the treatment was started. Patients with chronic diseases other than BD, patients diagnosed with malignancy (receiving chemotherapy or radiotherapy), patients with chronic inflammatory diseases (such as chronic obstructive pulmonary disease, sarcoidosis), patients with acute inflammation, and patients with ophthalmological problems other than BD uveitis were excluded from the study.

The control group was randomly selected from healthy volunteers aged between 23 and 56 years who were attending routine ophthalmological examinations and who did not have active inflammation. The selection process was computer-generated.

### 2.2. Biochemical measurement

Blood samples for routine biochemistry collected from study patients and controls were centrifuged at 4000 rpm for 15 minutes at +4°C after the tube was kept in an upright position for 10 to 20 minutes for clotting. The serum samples obtained were aliquoted and stored in a deep freezer at −80°C and kept there until analyzed.

In order to avoid daily variations, serum Tie-2, Ang-1, Ang-2, EphrinB2, EphB4, and endocan levels were measured on the same day. Serum Tie-2, Ang-1, Ang-2, EphrinB2, EphB4, and endocan levels were determined by enzyme-linked immunosorbent assay (ELISA) method using Human Tie-2 ELISA Kit (Bostonchem), Human ANG-1 ELISA Kit (Bostonchem), Human ANG-2 ELISA Kit (Bostonchem), Human EphrinB2 ELISA Kit (Bostonchem), Human EphB4 ELISA Kit (Bostonchem), and Human Endocan ELISA Kit (Bostonchem) according to the manufacturer’s instructions.

The procedures applied for the measurement were briefly as follows: microplates consisting of 96 wells coated with monoclonal antibodies against human Tie-2, Ang-1, Ang-2, EphrinB2, EphB4, and endocan were filled with serum and standard solutions obtained by serial dilutions with decreasing concentrations.

Tie-2, Ang-1, Ang-2, EphrinB2, EphB4, and endocan molecules present within the samples demonstrated binding to the coated antibodies. The process of purification entailed the elimination of unbound molecules through a rigorous washing procedure. Subsequently, a second antibody, specific for these molecules and labeled with biotin, was added to the wells. Following the process of 1 cycle of washing, the addition of the peroxidase enzyme, which had previously undergone binding with streptavidin, was conducted. The peroxidase enzyme present in the complex formed a bond with avidin, resulting in the oxidation of 3,3′,5,5′-tetramethylbenzidine that had been added to the medium. This reaction led to a color change, the intensity of which was directly proportional to the concentration of the molecules present in the samples. The process was then halted by the addition of acid to each well, thus arresting the reaction. The absorbances of each well were measured with a spectrophotometer at a wavelength of 450 nm. The concentration of Tie-2, Ang-1, EphrinB2, and EphB4 in each sample was calculated in ng/mL, while Ang-2 and endocan were measured in pg/mL. The data points for the calculation were determined using a standard curve prepared at decreasing concentrations. All biochemical measurements were performed in duplicate, and the mean value was used for analysis (intraclass correlation coefficient ≥ 0.90 for all markers). The ophthalmologists performing clinical evaluations and the laboratory personnel conducting biochemical analyses were blinded to each other’s results.

### 2.3. Statistical analysis

The SPSS 26.0 program was used for statistical analysis of the data. The Kolmogorov–Smirnov test was employed to analyze the conformity of the variables to a normal distribution. Descriptive analyses were performed using number and percentage for categorical variables, and mean and standard deviation for numerical variables. Independent comparisons of numerical variables in more than 2 groups were performed using a one-way analysis of variance test because the normal distribution condition was met in the groups. The relationships between numerical variables were analyzed by Pearson correlation analysis. Student *t* test was used as a statistical method for the statistical significance of the parameters fitting the normal distribution between 2 independent groups. The usability of the investigated serum molecules in the diagnosis of the disease was evaluated by the ROC analysis method. The cutoff point for variables considered to have diagnostic value was determined using the Youden index. *P* values <.05 were considered as statistically significant results.

## 3. Results

There were 20 (69%) male and 9 (31%) female patients in the first group with active Behçet uveitis, 6 (19.4%) male and 25 (80.6%) female patients in the second group without ocular involvement, and 19 (63.3%) male and 11 (36.7%) female patients in the completely healthy control group. There was a significant difference between the groups in terms of gender distribution (*P* < .001).

In the first group (active uveitis), patients were divided into panuveitis, anterior uveitis, and posterior uveitis according to examination findings. Among 29 patients, 22 (76%) had panuveitis, 5 (17%) had posterior uveitis, and 2 (7%) had anterior uveitis. In the panuveitis patient group, 3 patients had bilateral panuveitis attacks.

BCVA in patients with active Behçet uveitis was analyzed in 3 groups. Visual acuity of the patients ranged between 0.5 and 0.001. According to BCVA, patients were grouped as those with BCVA >0.1, between 0.1 and 0.05, and <0.05. BCVA in BD patients without ocular involvement and in the control group was complete (1.0; Table 1).

**Table 1:** BCVA of BD patients with ocular involvement according to involvement types.

Type of uveitis	BCVA	n (%)
Panuveitis (22 patients)	<0.05	10 (45.4)
	0.05–0.1	7 (31.8)
	>0.1	5 (22.7)
Posterior uveitis (5 patients)	<0.05	1 (20)
	0.05–0.1	2 (40)
	>0.1	2 (40)
Anterior uveitis (2 patients)	0.05	1 (50)
	0.1	1 (50)

BCVA = best corrected visual acuity, BD = Behçet disease.

The levels of endocan, EphrinB2, EphB4, Ang-2, and Tie-2 molecules were found to be significantly elevated in BD patients with active uveitis than in BD patients without ocular involvement, as well as in the control group. In addition, serum levels of these molecules were found to be significantly higher in BD patients without uveitis than in the healthy control group. Ang-1 was found to be significantly lower in the BD disease group with active uveitis than in the BD disease group without ocular involvement. It was found to be significantly lower in the BD group without ocular involvement compared with the control group (Table 2).

**Table 2:** Comparison of Ang-1, Ang-2, Tie-2, endocan, EphrinB2, and EphB4 levels of the groups.

Groups	Ang-1 (ng/mL)	Ang-2 (pg/mL)	Tie-2 (ng/mL)	Endocan (pg/mL)	EphrinB2 (ng/mL)	EphB4 (ng/mL)
Behçet + uveitis (+)	2.7 ± 1.7	716.7 ± 95.0	10.5 ± 3.7	463.0 ± 133.7	61.8 ± 12.5	13.3 ± 1.7
Behçet + uveitis (−)	4.1 ± 1.8	583.8 ± 178.6	7.4 ± 3.2	378.4 ± 130.1	46.8 ± 9.9	8.3 ± 1.6
Control group	5.4 ± 2.0	449.4 ± 116.2	4.6 ± 2.9	290.9 ± 100.9	27.5 ± 6.4	3.1 ± 1.4
Statistics						
1 vs 2 vs 3	*P* < .001	*P* < .001	*P* < .001	*P* < .001	*P* < .001	*P* < .001
1 vs 2	*P* = .014	*P* = .001	*P* = .002	*P* = .027	*P* < .001	*P* < .001
1 vs 3	*P* < .001	*P* < .001	*P* < .001	*P* < .001	*P* < .001	*P* < .001
2 vs 3	*P* = .03	*P* = .001	*P* = .006	*P* = .019	*P* < .001	*P* < .001

Data were given as mean ± SD.

Ang-1 = angiopoietin-1, Ang-2 = angiopoietin-2, SD = standard deviation, Tie-2 = tyrosine protein kinase receptor.

BD patients with ocular involvement were divided into 2 groups according to the examination findings: those with panuveitis and those with other forms of involvement (posterior or anterior uveitis). As the number of patients with posterior and anterior uveitis was small, these 2 groups were analyzed together as other involvement. Serum levels of endocan, EphrinB2, EphB4, and Tie-2 were found to be statistically significantly higher in patients with panuveitis than in those with other forms of involvement, whereas Ang-1 level was found to be statistically significantly lower in patients with panuveitis compared with patients with other involvement. Ang-2 was found to be higher in the group with panuveitis than in the group with other involvements, but there was no statistically significant difference (Table 3).

**Table 3:** Serum Ang-1, Ang-2, Tie-2, endocan, EphrinB2, and EphB4 levels in BD patients with active uveitis according to ocular involvement type.

Biomarkers	Panuveitis (n = 22)	Other types of inflammation (n = 7)	Statistical analysis value (*P*)
Endocan (pg/mL)	503.5 ± 127.5	335.7 ± 40.5	*P* = .002
Tie-2 (ng/mL)	12.0 ± 2.9	5.7 ± 0.9	*P* < .001
Ang-1 (ng/mL)	2.02 ± 0.96	5.14 ± 1.29	*P* < .001
Ang-2 (pg/mL)	734.3 ± 102.9	661.5 ± 19.1	*P* = .077
EphB4 (ng/mL)	13.9 ± 1.6	11.6 ± 0.3	*P* = .001
EphrinB2 (ng/mL)	66.2 ± 10.9	48.0 ± 4.7	*P* < .001

Data were given as mean ± SD.

Ang-1 = angiopoietin-1, Ang-2 = angiopoietin-2, BD = Behçet disease, SD = standard deviation, Tie-2 = tyrosine protein kinase receptor.

In the group with active uveitis, EphB4, Ang-2, and Tie-2 were negatively and highly significantly correlated with BCVA. EphrinB2 and endocan levels were negatively and moderately correlated with BCVA. A moderately significant positive correlation was identified between Ang-1 levels and the vision values of patients (Table 4).

**Table 4:** Correlation of serum Ang-1, Ang-2, Tie-2, endocan, EphrinB2, and EphB4 levels with BCVA in BD patients with active uveitis.

	BCVA	
	Correlation coefficient (*r*)	*P*
Endocan (pg/mL)	−0.658	*P* < .001
Tie-2 (ng/mL)	−0.701	*P* < .001
Ang-1 (ng/mL)	0.522	*P* = .004
Ang-2 (pg/mL)	−0.763	*P* < .001
EphB4 (ng/mL)	−0.774	*P* < .001
EphrinB2 (ng/mL)	−0.676	*P* < .001

Ang-1 = angiopoietin-1, Ang-2 = angiopoietin-2, BCVA = best corrected visual acuity, BD = Behçet disease, Tie-2 = tyrosine protein kinase receptor.

When all patient groups were evaluated together, the correlation coefficients and *P* values of the biomarkers are given in Table 5. The highest and significant correlation was found between EphrinB2 and EphB4. This was followed by a high level of significant correlation between Ang-1 and Ang-2. Only Ang-1 was negatively correlated with all markers. It was noteworthy that the correlations of the markers with each other were moderate or highly significant (Table 5).

**Table 5:** Correlation of Ang-1, Ang-2, Tie-2, endocan, EphrinB2, and EphB4 levels in all patient groups.

	Tie-2	Ang-1	Ang-2	Endocan	EphrinB2	EphB4
Tie-2						
*r*	–	−0.576	0.769	0.744	0.775	0.777
*P*	–	*P* < .001	*P* < .001	*P* < .001	*P* < .001	*P* < .001
Ang-1						
*r*	−0.576	–	−0.845	−0.646	−0.629	−0.656
*P*	*P* < .001	–	*P* < .001	*P* < .001	*P* < .001	*P* < .001
Ang-2						
*r*	0.769	−0.845	–	0.759	0.816	0.814
*P*	*P* < .001	*P* < .001	–	*P* < .001	*P* < .001	*P* < .001
Endocan						
*r*	0.744	−0.646	0.759	–	0.741	0.706
*P*	*P* < .001	*P* < .001	*P* < .001	–	*P* < .001	*P* < .001
EphrinB2						
*r*	0.775	−0.629	0.816	0.741	–	0.931
*P*	*P* < .001	*P* < .001	*P* < .001	*P* < .001	–	*P* < .001
EphB4						
*r*	0.777	−0.656	0.814	0.706	0.931	–
*P*	*P* < .001	*P* < .001	*P* < .001	*P* < .001	*P* < .001	–

Ang-1 = angiopoietin-1, Ang-2 = angiopoietin-2, Tie-2 = tyrosine protein kinase receptor.

**Figure 1.**
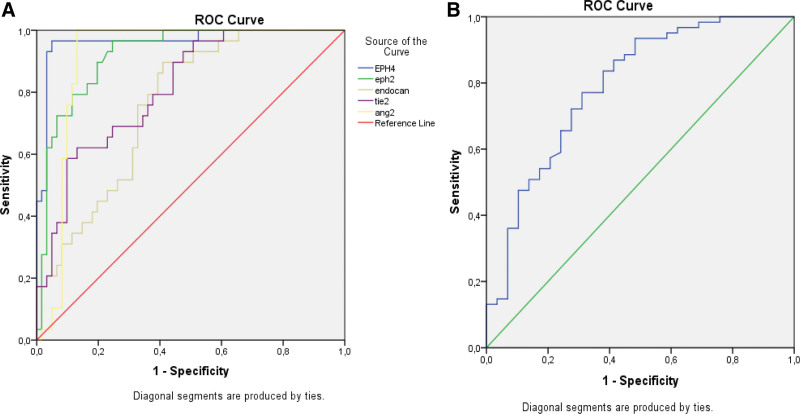
(A) ROC analysis of serum angiopoietin-2, Tie-2, endocan, EphrinB2, and EphB4 levels in all BD patients. (B) ROC analysis of serum angiopoietin-1 level in all BD patients. BD = Behçet disease, ROC = receiver operating characteristic, Tie-2 = tyrosine protein kinase receptor.

When we set the cutoff value for EphrinB2 at 44.54 ng/mL, its sensitivity and specificity were 96% and 85%, respectively, in differentiating patients with active uveitis from those without ocular involvement (area under the curve [AUC] = 0.921, *P* < .001). When we set a cutoff value of 10.97 ng/mL for EphB4, its sensitivity was 97%, and specificity was 95% (AUC = 0.965, *P* < .001). When the cutoff value for endocan was 334.93 pg/mL, its sensitivity and specificity were 90% and 59%, respectively (AUC = 0.760, *P* < .001; Fig. 1A).

When we set the cutoff value for Ang-2 at 631.62 pg/mL, sensitivity and specificity were 92% and 81%, respectively (AUC = 0.909, *P* < .001). When we set the cutoff value for Ang-1 at 3.31 ng/mL, sensitivity and specificity were 75% and 70%, respectively (AUC = 0.790, *P* < .001). For Tie-2, when the cutoff value was 9.87 ng/mL, sensitivity was 62%, and specificity was 87% (AUC = 0.811, *P* < .001; Fig. 1B).

## 4. Discussion

BD is a leading cause of blindness in our country and in countries with a high prevalence of the disease.^[18]^ Endothelial dysfunction, vascular inflammation, and neovascularization may play a crucial role in the pathophysiology of BD. Therefore, we studied Ang-1, Ang-2, Tie-2, endocan, EphrinB2, and EphB4 biomarkers that are associated with endothelial dysfunction, vascular inflammation, and neovascularization. In particular, we investigated the usefulness of these markers in determining ocular involvement and disease severity in patients with BD.

Ang-1 is a molecule that decreases endothelial cell permeability and shows anti-inflammatory properties. Conversely, Ang-2 is a molecule that becomes predominant in pathological conditions, triggering inflammation and leading to increased vascular permeability. Ang-1 and Ang-2 exert their effects by binding to the Tie-2 receptor on the vascular endothelium. Despite the heterogeneity of the BD manifestations, the prevailing pathology is characterized by vasculitis. Among the various manifestations of the disease, retinal vasculitis, which is observed in the majority of patients with ocular involvement, represents the most fundamental pathology. During an active uveitis attack, the release of cytokines and other inflammatory mediators results in a decrease in Ang-1 production, which protects the vascular structure and stabilizes the endothelium. Concurrently, Ang-2 production is triggered. The Ang-1/Tie-2 pathway is disrupted, and the Ang-2/Tie-2 pathway begins to function rapidly. The disruption of Ang-1 results in a loss of vascular integrity and the release of perivascular inflammatory infiltrates. On the other hand, Ang-2 perpetuates the inflammatory response and contributes to vasculitis by compromising endothelial integrity. The resultant increase in vascular permeability leads to the appearance of proteinaceous material and cells in the anterior chamber, causing anterior uveitis. The fern-like vascular leakage observed in posterior ocular involvement can be attributed to the disruption of the endothelial structure in retinal vessels. Ang-2 has been demonstrated to render retinal vascular structures more susceptible to angiogenic cytokines, such as vascular endothelial growth factor (VEGF), and inflammatory cytokines, including tumor necrosis factor-α and various interleukins.

Bassyouni et al showed that Ang-1 levels were found to be significantly lower in BD patients who exhibited vascular involvement, but increased in steroid-treated patients depending on the steroid dose. It has been suggested that Ang-2 release is suppressed and Ang-1 levels are increased in these patients due to the anti-inflammatory properties of the steroid.^[19]^ Choe et al found that patients diagnosed with BD demonstrated higher levels of Ang-1 in comparison with the healthy control group. However, the levels of Ang-1 and Ang-2 were comparable in BD patients with and without active involvement in terms of uveitis. Furthermore, it was determined that the levels of Ang-1 and Ang-2 were diminished in BD patients who exhibited ocular involvement in comparison with those who did not manifest such involvement. However, this decrease did not attain statistical significance.^[20]^

The present study found that levels of Ang-1 were reduced, while levels of both Ang-2 and Tie-2 increased, in patients diagnosed with BD compared with the control group. In patients with BD who exhibited active ocular involvement, lower levels of Ang-1 were observed, while higher levels of Ang-2 and Tie-2 were noted when compared with patients without such ocular involvement. In BD patients without ocular involvement, Ang-2 levels were higher and Ang-1 levels were lower compared with the control group, suggesting that subclinical inflammation persists in these patients and ocular complications may develop if this condition is not suppressed by treatment. In particular, in patients with ocular involvement, the Ang-2/Tie-2 pathway exhibited increased activity in comparison with the Ang-1/Tie-2 pathway.

Ephrin and Ephrin receptors are protein molecules with multiple subgroups on endothelium, epithelium, and leukocytes. In healthy tissues, there are normal levels of Ephrin and Ephrin receptors on endothelium and circulating cells. Leukocyte and endothelial adhesion is at a low level, while endothelial–endothelial adhesion is at a high level. Vascular endothelial integrity is preserved. In the early stages of inflammation, EphrinB2 ligand and EphB4 receptor production increase in endothelial tissue. The production of adhesion molecules on cell surfaces is triggered, and changes occur in the cytoskeleton. Thus, gaps develop in the endothelial barrier, and leaks occur from the vessel wall. In the progressive stages of inflammation, expression of EphB4 receptor and other Ephrin receptors on leukocyte and endothelial cell surfaces decreases, and adhesion of leukocytes and other blood elements to the vessel wall increases. Extravasation and transmigration from vascular structures occur.^[21]^

There are also many studies showing that EphrinB2 and EphB4 are involved in ocular angiogenesis, in which ischemia is involved in the etiopathogenesis of diseases such as retinal vein occlusions, diabetic retinopathy, and retinopathy of prematurity. Steinle et al demonstrated that EphrinB2 causes migration in retinal endothelial cells and is involved in retinal neovascularization.^[10]^ In our study, increased levels of EphrinB2 and EphB4 in patients with BD in comparison with a control group of healthy subjects support the existing literature. In addition, this increase is more prominent in BD with ocular involvement. In BD patients without active ocular involvement, higher levels of EphrinB2 and EphB4 proteins compared with the healthy control group may be a marker of a subclinical inflammatory condition. High levels of EphrinB2 and EphB4 in BD patients with active ocular involvement may be considered as an indicator of the inflammatory process. These molecules may have clinical significance in BD-related uveitis.

Endocan, an endothelial proteoglycan, has also been named as endothelial cell-specific molecule-1. Endocan plays an important role in endothelial cell activation and has an active role in angiogenesis. Endocan causes vascular dysfunction and transmigration by causing changes in cell adhesion. Various studies have been conducted on the role of the endocan molecule, which is known to trigger angiogenesis, in ocular neovascularization. Su et al reported that VEGF increased secondary to hypoxia in retinal, subretinal, and choroidal neovascularization, and VEGF triggered endocan production and caused new vessel formation.^[22]^

Balta et al found high serum endocan levels in BD patients with ocular and joint involvement. In addition, they showed that endocan levels were positively correlated with C-reactive protein, sedimentation, and disease activity. They argued that endocan may be responsible for vascular involvement in inflammation. In the ROC curve analysis, it was reported that the diagnostic sensitivity of the endocan molecule for BD was 75.8%, and its specificity was 80%.^[14]^ In our study, EphrinB2 and EphB4 were the markers with the best sensitivity and specificity in differentiating BD patients with ocular involvement from those without ocular involvement. Hassan et al showed that serum endocan levels were higher in patients with BD compared with the control group, and this elevation was more pronounced in BD patients with active involvement.^[13]^ In this study, serum endocan levels were found to be significantly elevated in patients diagnosed with BD compared with the control group. This elevation was particularly pronounced in patients with BD who also exhibited ocular involvement. The observation that endocan levels were higher in BD patients than in the healthy population, even in the absence of active ocular involvement, suggests the potential for endothelial dysfunction at the cellular level in this BD patient group, even in the absence of clinical findings.

When we analyzed the BD patients with active uveitis into groups with panuveitis, isolated anterior and isolated posterior involvement, EphrinB2, EphB4, Tie-2, and endocan levels were significantly higher in the panuveitis group, while Ang-1 levels were lower. The differences in the levels of these markers suggest that there may be an association between these molecules and panuveitis involvement with severe ocular inflammation. In addition, cells and proteinaceous material are seen in the anterior chamber as a result of endothelial dysfunction and increased vascular permeability in anterior uveitis and panuveitis. The leakage of retinal vascular structures in panuveitis and posterior uveitis can also be explained by the disruption of the vascular endothelial barrier. In summary, we think that these markers may play a role in these changes in the panuveitis of BD.

In order to question the clinical significance of our markers in BD-associated uveitis, we examined the relationship between the visual acuity of patients and the levels of the molecules. The findings revealed a significant negative correlation between the levels of Ang-2, Tie-2, EphrinB2, EphB4, and endocan in patients with BD uveitis, and a significant positive correlation with Ang-1 levels. Notably, EphB4 emerged as the most highly correlated marker. In patients with relatively high Ang-1 levels, uveitis manifests with a less severe presentation and does not result in a significant deterioration in visual acuity. Conversely, in cases of uveitis accompanied by severe inflammation, there is a notable increase in the levels of other markers, particularly Ang-2. This increase can result in a reduction in visual acuity.

The clinical relevance of identifying specific biomarkers for ocular pathology has been previously highlighted in several landmark studies. Zur et al identified disorganization of retinal inner layers as a structural biomarker in diabetic macular edema patients treated with dexamethasone implant, providing retina specialists with an objective tool to predict functional outcomes.^[16]^ Furthermore, next-generation anti-VEGF agents and longer-acting therapies for neovascular age-related macular degeneration have been evaluated in terms of their capacity to delineate and resolve pathological changes at the retinal level.^[23]^ These studies collectively demonstrate that objective biomarkers play a pivotal role in the early detection, severity assessment, and treatment monitoring of retinal diseases – a principle that equally applies to the ocular manifestations of systemic inflammatory conditions such as BD. In a similar manner, the serum biomarkers investigated in our study may provide clinicians with objective indicators of disease activity and ocular involvement in BD, complementing clinical examination findings and potentially improving diagnostic accuracy and treatment timing.

The findings of this study have potential clinical implications for the management of BD patients. Currently, the diagnosis and monitoring of ocular involvement in BD rely primarily on clinical examination and ophthalmological assessment. There are no validated serum biomarkers in routine clinical practice for detecting ocular involvement or predicting disease severity. Our results suggest that EphrinB2, EphB4, Ang-2, Tie-2, and endocan may serve as candidate biomarkers for this purpose. In particular, EphB4 demonstrated excellent diagnostic performance in distinguishing active uveitis from non-ocular involvement (AUC = 0.965, sensitivity = 97%, specificity = 95%), suggesting its potential utility as an objective marker of ocular inflammation. Furthermore, the observation that these markers were elevated even in BD patients without active ocular involvement indicates that subclinical endothelial dysfunction may precede clinical manifestation of uveitis. Early identification of such patients through biomarker screening could allow for timely therapeutic intervention, potentially preventing irreversible visual loss. Future studies with larger sample sizes are needed to validate the clinical utility of these markers and to evaluate their role in monitoring treatment response and guiding therapeutic decisions in BD-related uveitis.

This study had some limitations that may restrict the generalizability of the findings. It had a relatively small sample size, and the active uveitis group was heavily male (69%), while the group without uveitis was predominantly female (80.6%).

## 5. Conclusions

Based on the ocular involvement of BD, the role of Ang-1, Ang-2, Tie-2, EphrinB2, EphB4, and endocan molecules, which are effective on angiogenesis, vascularization, increase endothelial cell permeability, and thus play a role in inflammatory processes, was investigated. These markers have the potential to serve as novel biomarkers, allowing for the assessment of ocular involvement and disease severity in BD.

## Author contributions

**Conceptualization:** Betül Gökçek Barutcigil.

**Data curation:** Betül Gökçek Barutcigil, Esra Laloglu.

**Methodology:** Betül Gökçek Barutcigil, Emine Çinici, Esra Laloglu.

**Project administration:** Betül Gökçek Barutcigil, Emine Çinici.

**Resources:** Betül Gökçek Barutcigil.

**Validation:** Betül Gökçek Barutcigil.

**Investigation:** Emine Çinici.

**Supervision:** Emine Çinici.

**Formal analysis:** Esra Laloglu.

**Writing – original draft:** Betül Gökçek Barutcigil.

**Writing – review & editing:** Betül Gökçek Barutcigil.
